# Structural and Electronic Effect Driven Distortions in Visible Light Absorbing Polar Materials *A*Ta_2_V_2_O_11_ (*A* = Sr, Pb)

**DOI:** 10.1021/acs.jpcc.2c00469

**Published:** 2022-04-28

**Authors:** Artem A. Babaryk, Ievgen V. Odynets, Álvaro Lobato, Alaa Adawy, J. Manuel Recio, Santiago Garcia-Granda

**Affiliations:** † Department of Physical and Analytical Chemistry, University of Oviedo−CINN (CSIC), 33006 Oviedo, Spain; ‡ Taras Shevchenko National University of Kyiv, 64/13 Volodymyrska St., Kyiv 01601, Ukraine; § Malta-Consolider Team and Departamento de Química Física, Universidad Complutense de Madrid, 28040 Madrid, Spain; ∥ Unit of Electron Microscopy and Nanotechnology, Institute for Scientific and Technological Resources (SCTs), University of Oviedo, 33006 Oviedo, Spain; ⊥ MALTA-Consolider Team and Departamento de Química Física y Analítica, Universidad de Oviedo, 33006 Oviedo, Spain

## Abstract

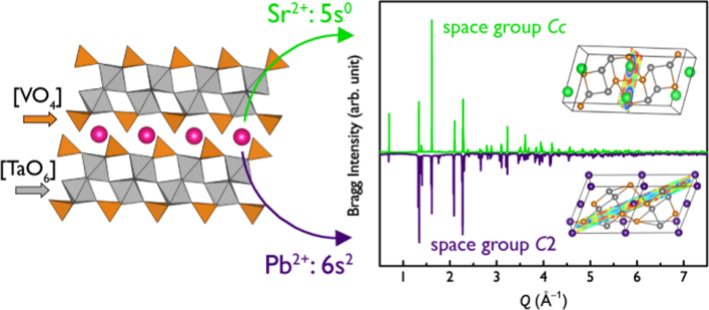

Complex vanadates of tantalum(V), such as *A*Ta_2_V_2_O_11_ (A = Sr, Pb), are rare and underrated materials, which have potential application domains that could be substantially expanded, mitigating the existing controversy on their atomic and electronic organization. Herein, we present a thorough structural examination combining synchrotron powder X-ray diffraction-aided distortion mode analysis with computational methods to study hettotypes of SrTa_2_V_2_O_11_ (STVO) and PbTa_2_V_2_O_11_ (PTVO). Being distinct from the perovskite family due to the presence of [VO_4_] groups, both compounds are polar dielectric materials with certain similarities to SBT and PBT Aurivillius phases. Applying the model of anions of metallic matrices to the analysis of electron localization functions calculated on top of as-established equilibrium structures helps retrace the effects in the Sr and Pb surroundings on the respective crystal packings of STVO and PTVO.

## 1 Introduction

The fast growth of global energy consumption calls for effective solutions to meet the continuously increasing global demands. The most promising sustainable source of clean energy is solar light since its colossal irradiation power may cover the world’s annual energy quota during a light day.^1^ The direct solar-to-electric energy conversion is possible in semiconductor materials under the absorption of incident photon matched by energy to the band gap (*E*_g_) with subsequent generation of an electron/hole pair, which is known as the photovoltaic effect. This principle is nowadays utilized in photovoltaic solar cells that consist of p- and n-type semiconductors coupled with a p–n junction to produce electric current through the migration of electrons and holes under a built-in electric field. The efficiency of a solar cell is confined within the Shockley–Queisser limit,^2^ putting a fundamental restriction on a single p–n junction solar cell. A possible solution to surpass the quoted barrier is the use of single-phase materials with a broken inversion symmetry. Such materials can generate a photovoltage exceeding *E*_g_ and steady photocurrent, which is basically known as the bulk photovoltaic effect (BPVE). Recently, Rappe et al. surveyed BPVE in polar materials, like hybrid and oxide perovskites, focusing on the shift current mechanism to formulate the principles based on the polar order, electronic structure, wave function, band gap, and density of state engineering.^3^ In terms of the shift current model, the low-dimensional structures (chain- or layer-like) ought to have larger light responses and are prospective candidate materials for photovoltaic applications.^4^ Oxides are a traditional domain for material research, providing rich opportunities for electronic structure engineering via cation or charge ordering of layered structures;^5^ thereby, the research space is continuously seeking for new BPVE materials.

The perovskite crystal structure has the fundamental importance for ABO_3_ oxides. The corner-sharing of [BO_6_] octahedra produces an infinite three-dimensional [BO_3_]_∞_ framework, providing a space for counter-cations (A) to reside. Despite having a simple structural organization, perovskites adopt a variety of derivative packing as a function of their cationic composition, ordering, and charge effects, also enabling a number of materials with polar direction induced by mechanical strain, temperature, or concentration factors.

**Figure 1:**
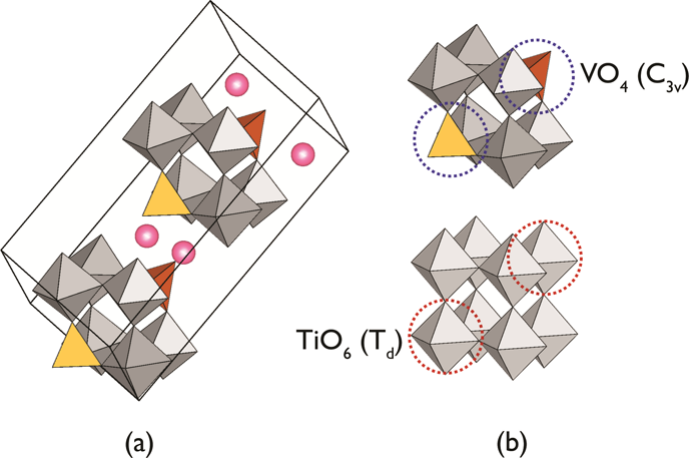
A perspective view of (a) [Nb_6_V_2_O_32_] secondary building units arranged within the cell of SrNb_2_V_2_O_11_ and (b) their comparison to [Ti_8_O_36_] for BaTiO_3_ (*Pm*-3*m*) perovskite.

Trunov et al. studied a group of oxides in the system *A*O–*B*_2_O_5_–*X*_2_O_5_ (*A* = Sr, Ba, or Pb; *B* = Nb or Ta; *X* = P or V), attributing them to a *palmeirite* type (K_2_Pb[SO_4_]_2_).^6^ Odynets et al. analyzed the case of a SrNb_2_V_2_O_11_ (SNVO) representative using experimental and computational methods to accurately determine the structure (Figure 1a) and showed that its polarity originates from the coherent off-center displacements of Sr and the tilts of O atoms of [Nb_6_V_2_O_32_] secondary building units.^7^ The latter resemble a perovskite structure upon a virtual replacement of [MO_6_] octahedra to [XO_4_] tetrahedra (Figure 1b). The shorter vanadyl bond (1.57–1.68 Å) within the trigonal pyramid-shaped (C_3v_) vanadate that possesses a dipole moment may result in a non-zero net polarization.^8^ A change of structural topology by incorporating polar secondary building units, like [VO_4_], into the framework could be a promising strategy for the design of polar photovoltaic oxides. It is also known that octahedra [*B*O_6_] (*B* = Ti^+4^, Zr^+4^, Nb^+5^, Ta^+5^, Mo^+6^, or W^+6^) typically feature polar distortion geometry due to the second-order Jahn–Teller effect.^9^

Therefore, polar structure engineering utilizing polar [*B*O_6_] building blocks expanded with [VO_4_] can yield interesting classes of materials beyond the traditional domain of perovskite oxides. In the present work, we focus our attention on *A*B_2_V_2_O_11_ [*A*: Sr, Pb].^6^ Although they are based on cations of *n*d^0^ and *n*s^2^, their respective band gaps fall within the visible part of the electromagnetic spectrum (2.01–2.45 eV) and can be red-shifted by being doped with Eu^3+^ for instance.^10^ Sr and Pb variants have a noncentrosymmetric (or polar) nature as confirmed by SHG experiments, showing a remarkably strong nonlinear optical response of 33–50% LiNbO_3_.^11^ This fact also opens the interesting possibility of using both vanadates in dielectric applications.

In this work, we explore how distortions in layered [Ta_2_V_2_O_11_] frameworks are unexpectedly balanced in two different crystalline structures when Sr and Pb counter-cations are incorporated. By using high-resolution X-ray diffraction supported by electron microscopy and computational simulations, we could account for the changes in the geometry and electronic structure influenced by the Sr^2+^ and Pb^2+^ cations and provide a reasonable explanation of these differences. We believe that the outcomes from the present comparative analysis of the structures, at both the structural and electronic levels, will contribute to the field of either BPVE or dielectric perspective materials. The focus in this contribution is on the characterization of properties related to the potential use of both compounds as BPVE materials, whereas the SHG response will be the subject of subsequent investigations.

## 2 Methods

### 2.1 Synthesis and Characterization of Powders

Crystalline powders of SrTa_2_V_2_O_11_ (STVO) and PbTa_2_V_2_O_11_ (PTVO) were prepared by a conventional solid-state reaction according to the following reactions:
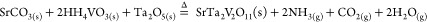

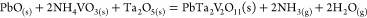


To decipher the optimal temperatures of synthesis, thermogravimetric (TG) scans of the reacting blends were performed coupled with differential thermoanalysis (DTA) and *ex situ* powder X-ray diffraction measurements. Full details of those investigations are summarized in the Supporting Information (see also Tables S1 and S2 and Figures S3 and S4). A detailed description of samples of both compounds including absorption spectra and band gaps is reported in ref (11).

#### 2.1.1 SrTa_2_V_2_O_11_

Initial components SrCO_3_ (strontianite, 99%, Reakhim, USSR), Ta_2_O_5_ (>99.9%, Alfa Aesar, UK), and NH_4_VO_3_ (99%, Reakhim, USSR) were mixed in a molar ratio 1:1:2 and wet-milled under added 2-propanol (99.9%, Macrochem, Ukraine) as a liquid active medium for 30 min in an agate mortar. The starting pulverized blend was pressed into a pellet (*P* = 105 N m^–2^, Ø = 20 mm, *d* = 2 mm) and heated at a muffle furnace for 6 h at 650 °C. Then, the sample was reground, re-pressed, and finally calcined at 800 °C for 12 h.

#### 2.1.2 PbTa_2_V_2_O_11_

Initial components PbO (99%, Reakhim, USSR), Ta_2_O_5_ (99.9%, Alfa Aesar), and NH_4_VO_3_ (99%, Reakhim, USSR) were mixed in a molar ratio 1:1:2 and wet-milled under added 2-propanol (99.9%, Macrochem, Ukraine) as a liquid active medium for 30 min in an agate mortar. The starting pulverized blend was pressed into a pellet (*P* = 105 N m^–2^, Ø = 20 mm, *d* = 2 mm) and heated at a muffle furnace for 6 h at 300 °C. Then sample was reground, re-pressed, and finally calcined at 700 °C for 24 h.

### 2.2 Synchrotron X-ray Powder Diffraction (SPXRD)

High-resolution synchrotron powder diffraction data were collected at the same temperature on beamline 11-BM at the Advanced Photon Source at Argonne National Laboratory, USA,^12^ using an average wavelength of 0.41570 Å. Discrete detectors covering an angular range from −6 to 16° in 2θ were scanned over a 34° 2θ range, with data points collected every 0.001° in 2θ and at a scan speed of 0.01° s^–1^. The precalculated amount of the target compound was diluted with X-ray diffraction checked amorphous silica powder and sealed in a standard 0.4 mm Kapton capillary with plasticine.

### 2.3 Rietveld Refinements

The SPXRD pattern of STVO is unambiguously indexed in a monoclinic cell setting, the space group *Cc*, in contrast to the previously proposed supergroups *R*3*m* or *C*222_1_.^11,13^ Fractional atomic coordinates were adopted from our recent study on the isostructural SrNb_2_V_2_O_11_ compound^7^ for the initial model. Background contribution was accounted for by selecting fixed points manually, fitted by shifted Chebyshev polynomials function (up to the 30th order) with refined terms. The scale factor, unit cell parameters, detector zero shift, and isotropic contributions to size and strain broadening were refined. Ta, V, Sr, and O positions were relaxed consecutively from the heaviest to the lightest sort of atom. The phenomenological model of microstrain^14^ was employed to better account for the anisotropic strain broadening effect observed for the high-resolution PXRD data set. A small portion [0.0355(3) wt %] of unreacted Ta_2_O_5_ has been detected by dominant *d*_200_ = 2.064 Å reflection,^15^ and the cell parameters and Lorentzian term of size parameter were refined only.

Inspection of systematic absences for PTVO shows that *h*0*l*: *h,l* = 2*n* and 00*l*: *l* = 2*n* do not extinct, which means that the symmetry reduction to *C*2 or *Cm* space groups is equiprobable. The structure has been solved by direct methods using the EXPO2014 software and refined using the scheme as described above for SrTaV_2_O_11_. A trace [0.0089(11) wt %] of Ta_9_VO_25_ was detected and included as a secondary phase to improve the quality of refinement.^16^ The final plot of Rietveld refinement for STVO and PTVO is given in Figure S2.

### 2.4 High-Resolution Transmission Electron Microscopy

The [high-resolution] transmission electron microscopy ([HR]TEM) studies were performed on a JEOL JEM-2100F transmission electron microscope operating at an accelerating voltage of 200 kV and equipped with a field-emission gun and an ultra-high-resolution pole piece that provided a point resolution better than 0.19 nm. The micrographs were taken using a CCD camera (Gatan 14-bit Orius SC600). This microscope was used to perform TEM, HRTEM, and selected area electron diffraction (SAED). Fine powder of every sample was dispersed in ethanol, shortly sonicated, sprayed on a Lacey-carbon-on-copper grid (200 mesh, EM science), and then allowed to air-dry. Then, the dried grid was mounted on a JEOL single-tilt holder. Acquiring, processing, and analyzing of all micrographs were performed using the Gatan Digital Micrograph software.

### 2.5 Periodic Boundary Density Functional Theory Calculations

Periodic boundary density functional theory (PB-DFT) calculations were carried out using the projector-augmented wave method, as implemented in the Vienna Ab initio Simulation Package (VASP).^17,18^ Initial coordinates of atoms in the unit cell were taken from PXRD refined crystal structures and used for further optimizations. Generalized gradient approximation of DFT in Perdew–Becke–Ernzerhof parametrization optimized for solids was applied to account for electron exchange and correlation.^19^ The valence electrons considered for each atomic species are as follows: 5p^6^5d^4^6s^1^ for Ta, 3s^2^3p^6^3d^4^4s^1^ for V, 2s^2^2p^4^ for O, 4s^2^4p^6^5s^2^ for Sr, and 5d^10^6s^2^6p^2^ for Pb. The plane-wave cutoff energy was set to be 520 eV. Brillouin zone sampling of electronic states was performed on 5 × 5 × 3 Monkhorst–Pack grid. The structure was fully relaxed by the residual minimization method–direct inversion in the iterative space,^20^ after which the residual forces were converged to <10^–2^ eV Å^–1^ and free of Puley stress. The lattice dynamics of optimized structures has been studied by the density functional perturbation theory.^21,22^ The electron density and the electron localization function (ELF) scalar fields were topologically analyzed by means of the CRITIC2 code.^23^

## 3 Results and Discussion

### 3.1 Packing Effects of the STVO and PTVO Crystal Structures

**Figure 2:**
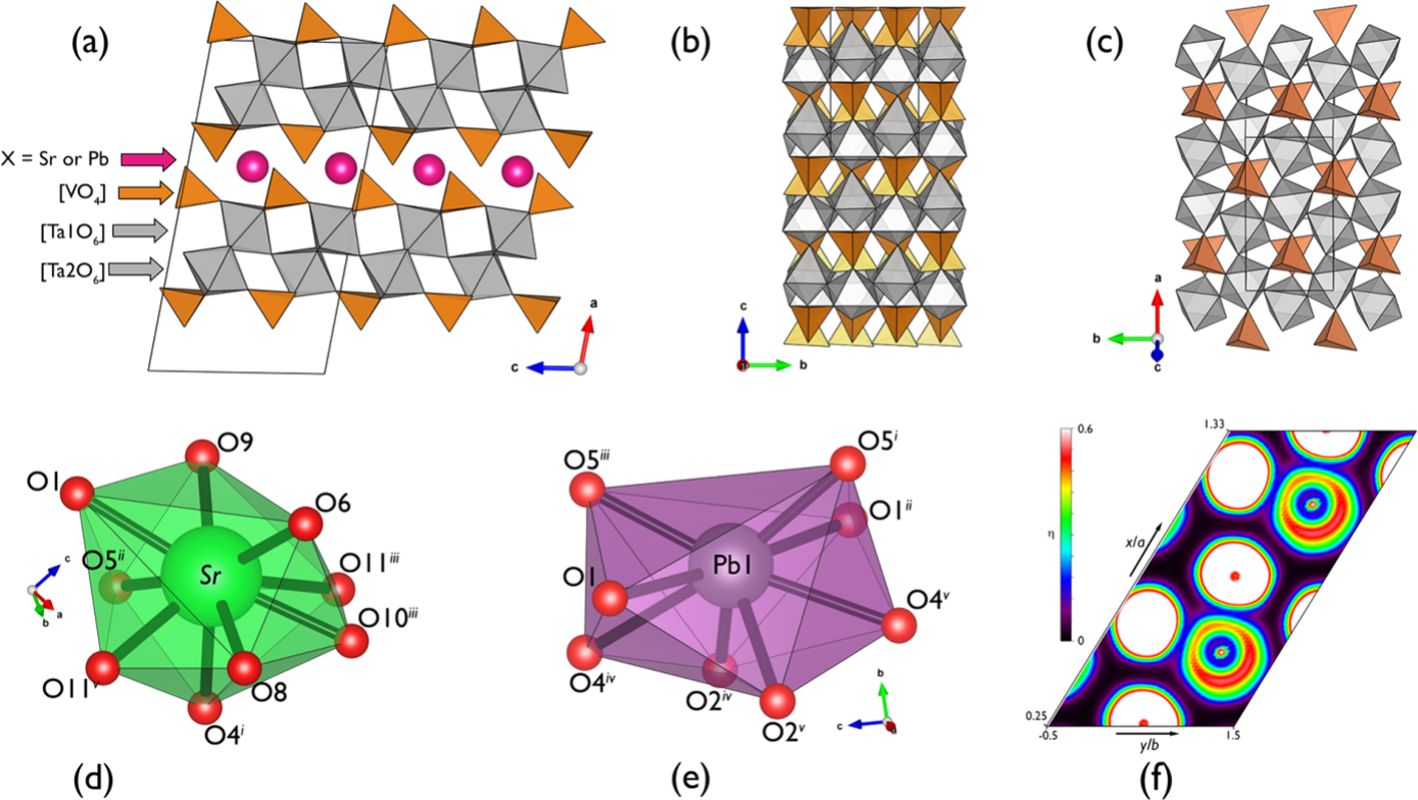
(a) The common projection for the *X*Ta_2_V_2_O_11_ (X = Sr or Ta) crystal structures shows them along the *b* axis; pseudo-hexagonal arrangement of [Ta_2_V_2_O_11_] sublattices of (b) STVO and (c) PTVO; (d, e) local coordination environment of the Sr atom at STVO [symmetry codes to generate equivalent positions of atom: (*i*) *x*, 1 – *y*, 1/2 + *z*; (*ii*) −1/2 + *x*, 1/2 + *y*, *z*; (*iii*) 1/2 + *x*, 1/2 + *y*, *z*; (*iv*) 1/2 + *x*, 1/2 – y, −1/2 + *z*] and Pb at PTVO [symmetry codes to generate equivalent positions of atom: (*i*) *x*, *y*, −1 + *z*; (*ii*) – *x*, *y*, −*z*; (*iii*) – *x*, *y*,1 – *z*; (*iv*) −1/2 + *x*, −1/2 + *y*, *z*; (*v*) 1/2 – *x*, −1/2 + *y*, −*z*] structures; (f) least-squares isosection (η = 0.6) of the electron localization function calculated through the Pb and O atoms of the DFT-optimized PTVO model (primitive cell setting, **a**_prim_ = 1/2(**a**_conv_ + **b**_conv_); **b**_prim_ = 1/2(**a**_conv_ – **b**_conv_); **c**_prim_ = −**c**_conv_).

While STVO crystallizes iso-structurally to SNVO, the half-volume unit cell was found for PTVO. Remarkably, the highest possible symmetry *C*2 (#5 in ITC) was detected for PTVO with (*hkl*) triplets with *l* = 2*n* + 1, in contrast to the non-isomorphic space group *Cc* (#9 in ITC), which is a common choice for STVO and SNVO. The experimental SPXRD patterns were analyzed with the Rietveld profile method, and the structural stability of this result was confirmed by ground-state calculations and phonon frequencies at the Γ(0, 0, 0) point, which are discussed below. The details of the refinements are summarized in the Tables S3–S6. Both structures feature [Ta_2_V_2_O_11_] layers; those consist of vertex-sharing [TaO_6_] and [VO_4_] polyhedra, spreading coplanar to (110), as shown on Figure 2a. The bond distances fall into groups on the criteria 1.96 < *d*_Ta–O_ > 1.97 Å (″[3 + 3]″) and 1.56 < *d*_V–O_ > 1.58 Å (″[3 + 1]″), adopting coordination polyhedra of pseudo-*C*_3*v*_ symmetry. Indeed, the unit cell transformation **a**_h_ = (−1/2**a**_m_ + **c**_m_) ≈ (1/2**a** – **b**_m_), **c**_h_ = 3**c**_m_ produces a pseudohexagonal unit cell of **a**_h_ ≈ 5.49 Å, **c**_h_ ≈ 27.22 Å (Figure 2b,c) reported to be common for *AB*_2_*X*_2_O_11_ (*A* = Sr, Ba, or Pb; *B* = Nb or Ta; and *X* = P or V) structures.^6^ Each [Ta_2_V_2_O_11_] layer is terminated with apical O atoms of [VO_4_], which are not engaged into a linkage with the adjacent [TaO_6_]. The calculated bond valence^24^ (1.77 < BVS < 2.14, see Tables S4 and S6) and the Hirshfeld charge^25^ difference Δ*q*_e_ = 1.31 (STVO) and Δ*q*_e_ = 1.168 (PTVO) (see Tables S7 and S8) for terminal V–O suggest an intermediate state between ″V–O″ (*d*_calc_ = 1.803 Å) and ″V=O″ (*d*_calc_ = 1.546 Å), revealing a strong covalent character of the bonds. The stacking of the layers along [001] provides a space for Sr or Pb atoms to reside. The former is enclosed with nine [8 + 1] oxygen atoms at a distance of *d*_max_ < 3.15 Å (Figure 2d). The Pb environment is more compact (polyhedral volume is 28.27 versus 35.37 Å^3^ for SrO_9_) and consists of the six nearest (2.47 < *d*_max_ < 2.70 Å) and two distant contacts (*d*_Pb–O_ = 3.063 Å) (Figure 2e). Taking into account the strong resemblance of ionic radii of ninefold and eightfold coordinated Sr^2+^ and Pb^2+^ ions (1.31 and 1.29 Å according to Shannon’s classification^26^), purely electronic effects may explain the difference in packing. The stereochemical impact of the Pb 6*s*^2^ lone electron pair is typical for a number of inorganic compounds, including ferroelectric oxides.^27^ To explore this effect, we used the electron localization function (ELF), which is a straightforward quantity mapped onto the calculated electron density at the ground state. An exhaustive topological analysis of this scalar field is presented below. In Figure 2f, we anticipate how the ELF depicts the lone electron pair as a lobe around Pb atoms. This allows one to interpret the coordination of lead atoms as ″[8 + lone pair]″ resembling the ninefold coordination (tricapped triangular prism) shown in other lead compounds such as PbCl_2_ and PbO_2_.^28^

### 3.2 HRTEM Analysis for *X*Ta_2_V_2_O_11_ (X = Sr or Pb) Crystal Structures

The TEM investigations showed that both *A*Ta_2_V_2_O_11_ have the morphology of nanolayered structures with dimensions varying between tenths to few hundreds of nanometers (Figure S3a,b). The selected area electron diffraction (SAED) for numerous nanolayers of both structures shows a typical ring pattern and reported some reflections consistent with those specified using SPXRD (Figure S3c,d).

**Figure 3:**
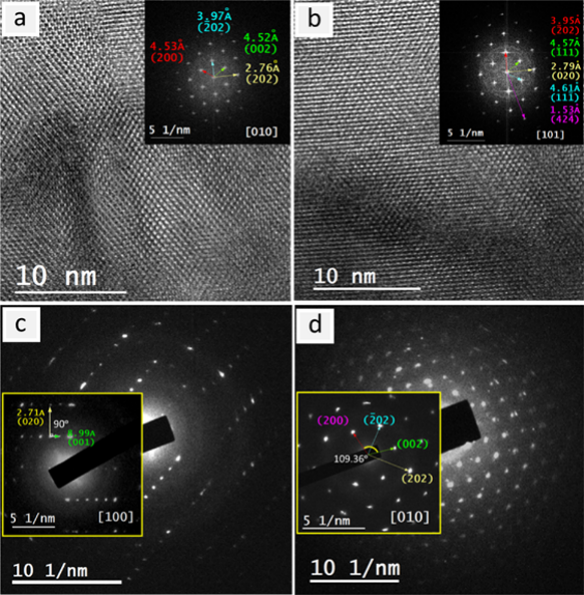
Electron microscopy of PbTa_2_V_2_O_11_ nanolayers: (a, b) HRTEM images and their indexed FFT (insets) in the zone axes [010] and [101]; (c, d) SAED patterns and their indexing (insets) in the zone axes [100] and [010].

**Figure 4:**
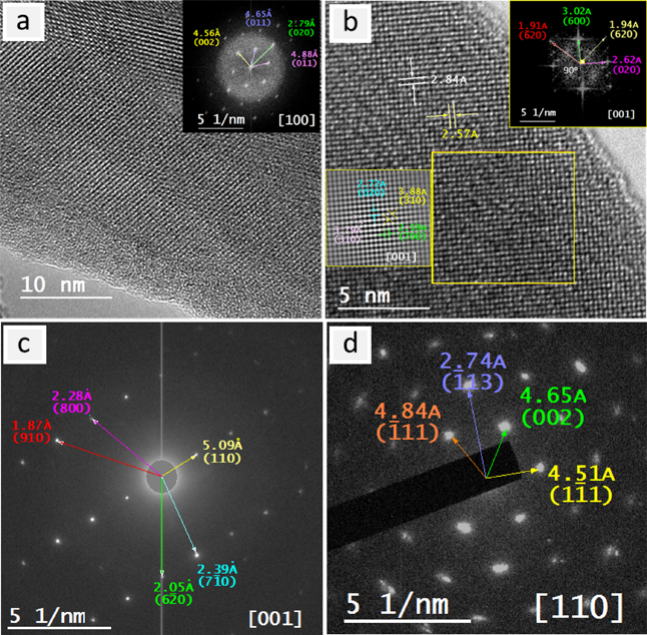
Electron microscopy of SrTa_2_V_2_O_11_ nanolayers: (a) HRTEM image and its indexed FFT (inset) in the zone axis [100], (b) HRTEM image and autocorrelation and FFT (insets) for the area indicated by the yellow square in the zone axis [001]; (c, d) SAED patterns and their indexing in the zone axes [001] and [110].

**Figure 5:**
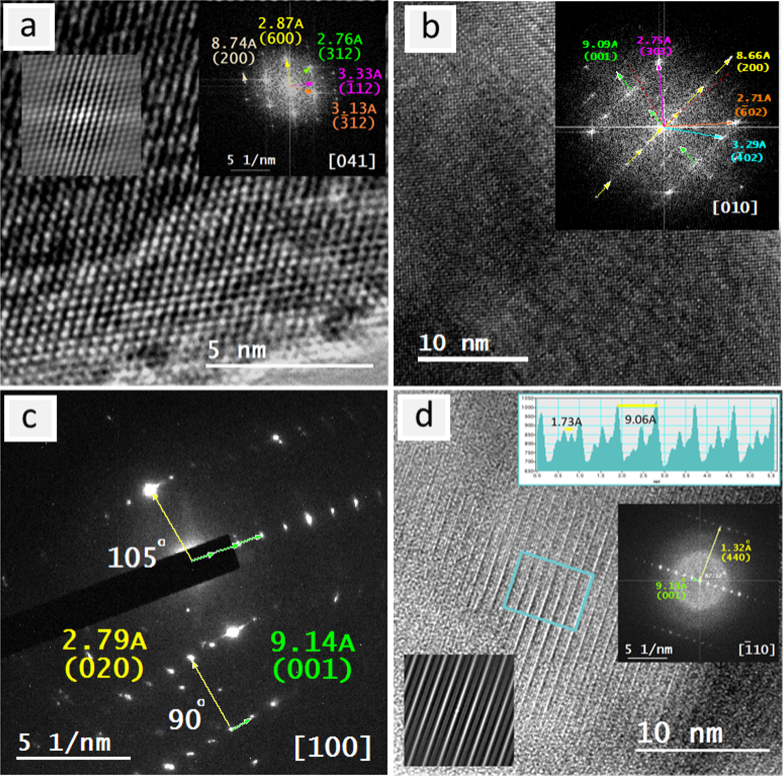
Electron microscopy of SrTa_2_V_2_O_11_ nanolayers revealing some planar defects: (a) HRTEM image and its indexed FFT and autocorrelation pattern (insets) in the zone axis [041] showing the streaking of the spots attributed to the (100) plane. (b) HRTEM image and its indexed FFT (inset) in the zone axis [010]. The red dashed lines indicate the original positions of the planes (001) and (200) before their rotation. (c) SAED pattern and its indexing in the zone axis [100] showing the short-range directional ordering of the plane (001). (d) HRTEM image and its autocorrelation and indexed FFT in the zone axis [1̅10] (insets) showing the appearance of (001). An area profile (inset) for the selected green square shows the d_*hkl*_ corresponding to (001) with five in between d_*hkl*_’s that would correspond to (005).

The HRTEM inspection showed the lattice fringes on both *A*Ta_2_V_2_O_11_ nanolayers in several directions, demonstrating the crystallinity of the nanolayers (Figures 3a,b and 4a,b). Although there are slight differences in the measured d_*hkl*_ values, most probably due to the electron beam damage, the indexing of their FFTs and of other SAED patterns has confirmed the structural information determined by relying on SPXRD with some contractions in the determined dimensions (Figures 3c,d and 4c,d and Figures S4 and S5). Compared with PbTa_2_V_2_O_11_, SrTa_2_V_2_O_11_ shows less crystallinity with more deterioration when subjected to the electron beam. According to the determined space groups based on SPXRD (*C1c1*), some forbidden reflections {(011), (01̅1), 41̅1), (401), (001), and (005)} were observed (Figures 4 and 5d and Figure S5) for STVO. Some of these reflections were expected to be a result of multiple elastic scattering that is usually known in electron diffraction.

In the fast Fourier transforms of HRTEM micrographs for several nanolayers of SrTa_2_V_2_O_11_ in the zone axes [010] and [041], a unidirectional streaking appeared in the diffraction spots corresponding to the plane (200) (Figure 5a,b). Most probably, these streaks are due to the presence of some planar defects (stacking faults) altering the stacking sequence of the plane (200), which should be normal to (020) (Figure 4a). However, in the zone axis [001], the plane (020) appears without any odd pattern, while the plane (200) could not be observed but rather its multiplicities (600) (Figure 4b). On the other hand, in some SAED patterns in which the zone axis [100] could be visualized, due to the appearance of the two planes ((001) and (020)), a short-range directional ordering of the plane (001) could be observed with an occasional tilt up to an angle of 15° (Figure 5c). This angle is around the same as that measured for the change in the direction of planes (001) and (200) in the zone axis [010] (Figure 5b). The same plane (001) was also observed in the [1̅10] but rather as a result of the failure of the (005) plane to grow horizontally in the same direction (Figure 5d).

### 3.3 Symmetry-Adapted Distortion Mode Analysis

**Figure 6:**
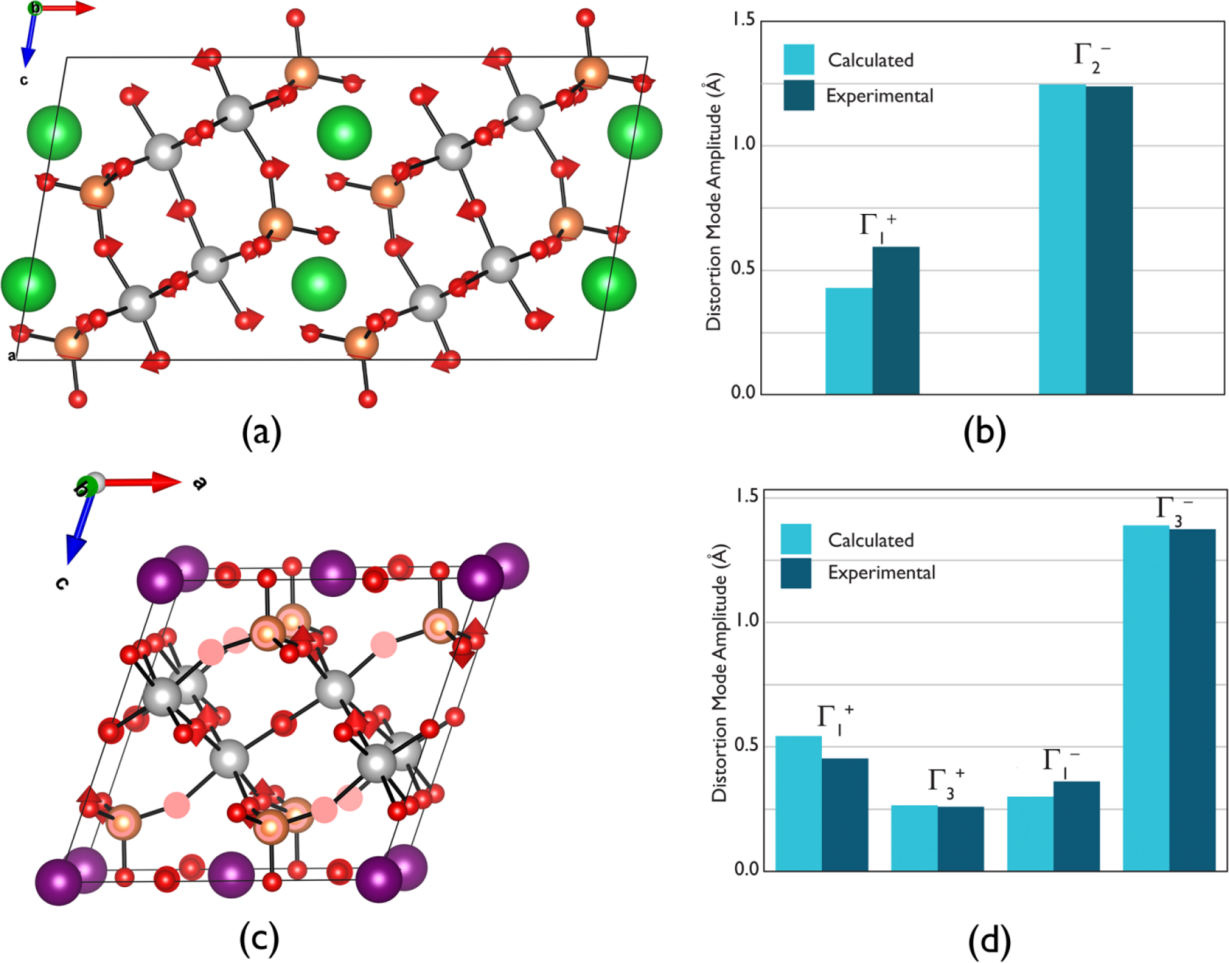
Visualization of atomic displacements under (a) Γ_2_^–^ primary distortion of STVO and (c) Γ_3_^–^ distortion of PTVO structures. Oxygen, vanadium, tantalum, strontium, and lead atoms are depicted as red, orange, gray, green, and violet spheres. (b, d) Comparative diagrams of distortion mode amplitudes obtained from analysis of DFT-optimized supergroup/subgroup models and direct SPXRD refinements.

Symmetry-adapted mode analysis is a powerful tool to facilitate the understanding of structural distortions decomposing it to a finite number of modes under the framework of the crystallographic group–subgroup relationship.^29^ This allows one to quantitatively estimate the contribution of each mode to the overall distortion of a structure. The search of supergroup variants of STVO and PTVO was carried out using the PSEUDO routine at the Bilbao Crystallographic Server.^30^ As-found models were relaxed to their ground-state equilibrium geometries using the same computational procedures as for Rietveld refined structures, ensuring stress-free conditions for further considerations. Experimental distortion mode amplitudes were obtained from refined SPXRD data. The resulting unit cell parameters and crystal coordinates are given in Tables S9 and S10. Distortion modes were determined using the ISODISTORT program.^31,32^ In the case of STVO, one can distinguish that the primary mode described with irreducible representation (irrep) Γ_2_^–^ of amplitude 1.199 Å breaks the inversion symmetry of the parental framework. The corresponding atomic displacement is visualized in Figure 6a,b. TaO_6_ octahedra and VO_4_ tetrahedra perform out-of-phase rotations in the (*ab*) plane affecting rather interpolyhedral geometry parameters. Typically, a weaker secondary mode is necessarily coupled to the primary one, and in the present case, the corresponding amplitude is of 0.429 Å (irrep Γ_1_^–^). One can see correlated rotations of oxygen atoms in the equatorial plane of TaO_6_ and around the (pseudo-threefold)-axis of VO_4_ in the plane holding the **b** vector. This may be interpreted as a rigid-unit mode (RUM), which uses to appear for oxide materials built of a octahedral–tetrahedral framework.^33^ For PTVO polar distortion Γ_3_^–^ of a 1.391 Å is associated with shifts of oxygen atoms of TaO_6_ octahedra and VO_4_ tetrahedra normally with respect to the [100] direction and along [010] (Figure 6c,d).

### 3.4 Electronic Band Gaps and Structure

**Figure 7:**
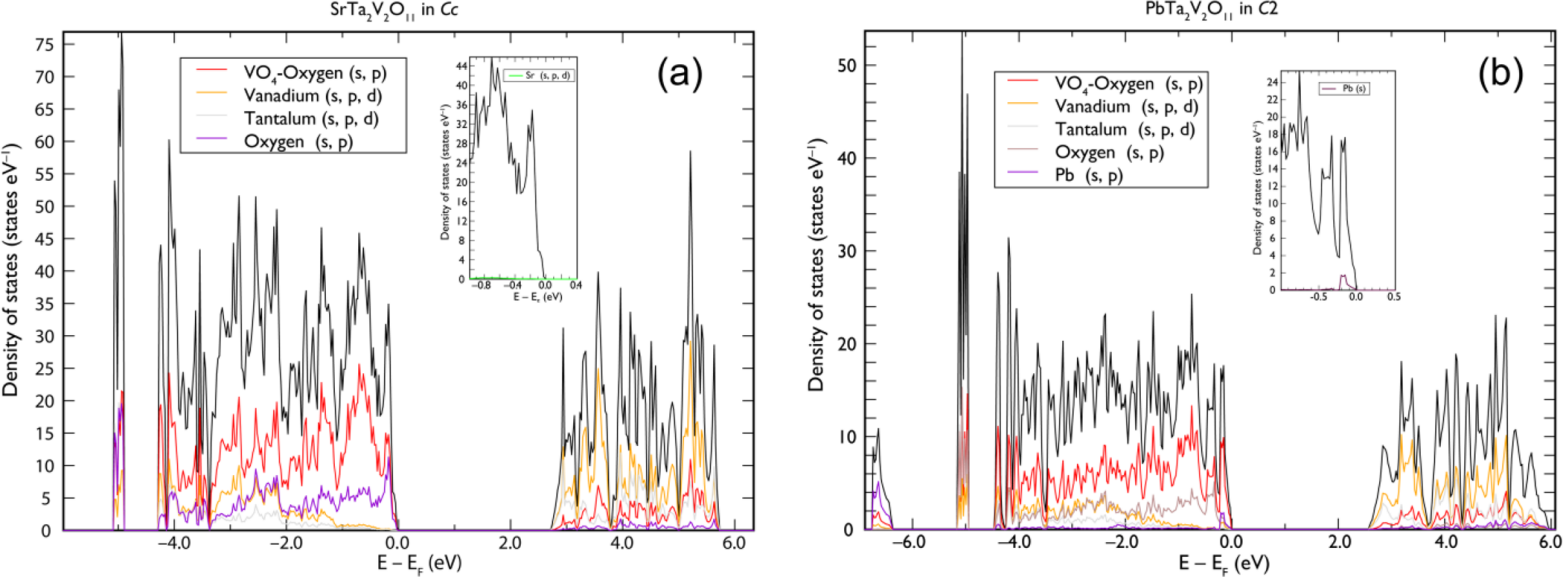
Total and partial density of states plots for STVO (a) and (b). Note the difference in the orbital contribution of Sr and Pb atoms as shown in the inset at each panel.

At the chosen level of theory, both STVOs appear to be the direct band gap semiconductors of 2.67 eV, which is a useful characteristic for potential optoelectronic applications. The band gap of PTVO is of indirect type transition with the energy of 2.55 eV. Although spin–orbit effects may alter quantitatively (reduction) or qualitatively (change from direct to indirect) the band gap as discussed by Rao et al.,^34^ we note that these relativistic effects in PTVO have an almost negligible impact (<0.1 eV) on the resulting indirect band gap value. A deeper understanding of the electronic building of titled compounds is possible, referring to the partial density of states (PDOS) plots (Figure 7). The 2s and 2p states of the oxygens belonging to VO_4_ groups dominate clearly across all valence band (VB) zones, while V 3s + 4s, 3p, 3d and Ta 5p, 5d, 6s states are notable deeper in the VB zone, and their impact significantly decreases close to the Fermi level. The oxygen atoms, those that are not involved in bonding with Ta atoms, contribute less to all states but the top of the VB zone. In contrast, the conduction band (CB) zone is rich with the empty states of vanadium and tantalum atoms slightly hybridized with those of oxygen atoms. Most of the features are found common for both STVO and PTVO considering the partial (atom- and angular-resolved) density of states corresponding to the atoms of anionic [Ta_2_V_2_O_11_] sublattices. However, a small but significant difference exists between structures when looking at the contributions of Sr and Pb atoms. While the former marks an appearance of a flat distribution of d-states at lower energies of the VB zone, the latter shows a pronounced structured group of 6s-states close to the Fermi level.

### 3.5 Dielectric Properties

Dielectric permittivity and Born effective charges (BECs) are tensor quantities important for the identification of the long-range dipolar contribution to phonon properties of a polar semiconductor or an insulator. Tables S11 and S12 sum up the present results obtained with the aid of the DFPT calculations. The total dielectric permittivity (ε_avr_) is nearly the same for STVO and PTVO, but as smaller as twice versus SNVO. Reference experimental figures are 16.5 and 8.7 in the case of STVO and PTVO, although the corrections to measured densities of ceramic materials are unreported. Here we note that, applying a similar methodology of study to SNVO leads to excellent agreement of experimental and theoretical values.^7,11^ Typically, BECs may be significantly higher than their expected formal charges for the ferroelectric phase of a material. For instance, Z*_avg_ (Ta) reaches 8.34 (8.41) for STVO (PTVO), which is a quantity comparable to that reported for perovskite-type AgTaO_3_ (8.87) but higher than that found for the Ruddlesden–Popper Li_2_CaTa_2_O_7_ (6.12).^35,36^ Anomalously high equatorial and axial components of dynamic charges assigned to O2, O3, and O7 are equal to −6.40, −5.76, and −5.71. Such observation correlates to the shortening of corresponding Ta–O bonds to 1.84–1.89 Å (see Table S4), indicating that some portion of covalency may be present within it. Remarkably, BECs of vanadium atoms are very close to their expected formal charges. In a contrast, dynamic charges of Sr and Pb surmount nominal values, showing closer correspondence to dielectric properties of SrTiO_3_^37^ and PbTiO_3_^38^ classical ferroelectric materials at their ground state.

### 3.6 General Discussion

The strong segregation of bond distances revealed by X-ray structure analysis may help to re-establish the actual structural moiety formula that is better expressed as *X*Ta_2_O_3_[VO_4_]_2_ (*X* = Sr or Pb). Not only is the given transcription essential for the formal systematization purposes, but also it helps to disclose functional parts of the structure. Referring to the present results of DFPT calculations, the [*X*Ta_2_O_3_] array of atoms may be considered as the most polarizable part of the structure. On the other hand, [VO_4_] groups play a dominant role in forming the VB zone and lowering the energy of the main optic transition. In comparison to the Aurivillius family of polar oxides, (Bi_2_O_2_)^2+^(*A*_*m*–1_B_*m*_O_3*m*+1_), complex structural characteristics of present materials show a certain parallelism. For instance, the main charge transfer is possible for SrBi_2_Ta_2_O_9_ (Bi_2_O_2_[SrTa_2_O_7_], SBT) due to the efficient hybridization of Bi 6p and O 2p orbitals within the [Bi_2_O_2_]^2+^ slab substructure, while in the present case, this function is prioritized by V 3d states in the CB zone. This effect is known for the SrBi_2_Nb_2_O_9_ doped with vanadium (up to 0.5%) and shown to reduce the band gap energy from 3.2 to 2.7 eV.^39^ According to Miura,^40^ Pb 6s^2^ orbitals in PbBi_2_Nb_2_O_9_ are found at the semi-core state region, which is in a sharp contrast to PTVO, where the same sort of atoms participates in the formation of the top of the VB zone.

**Figure 8:**
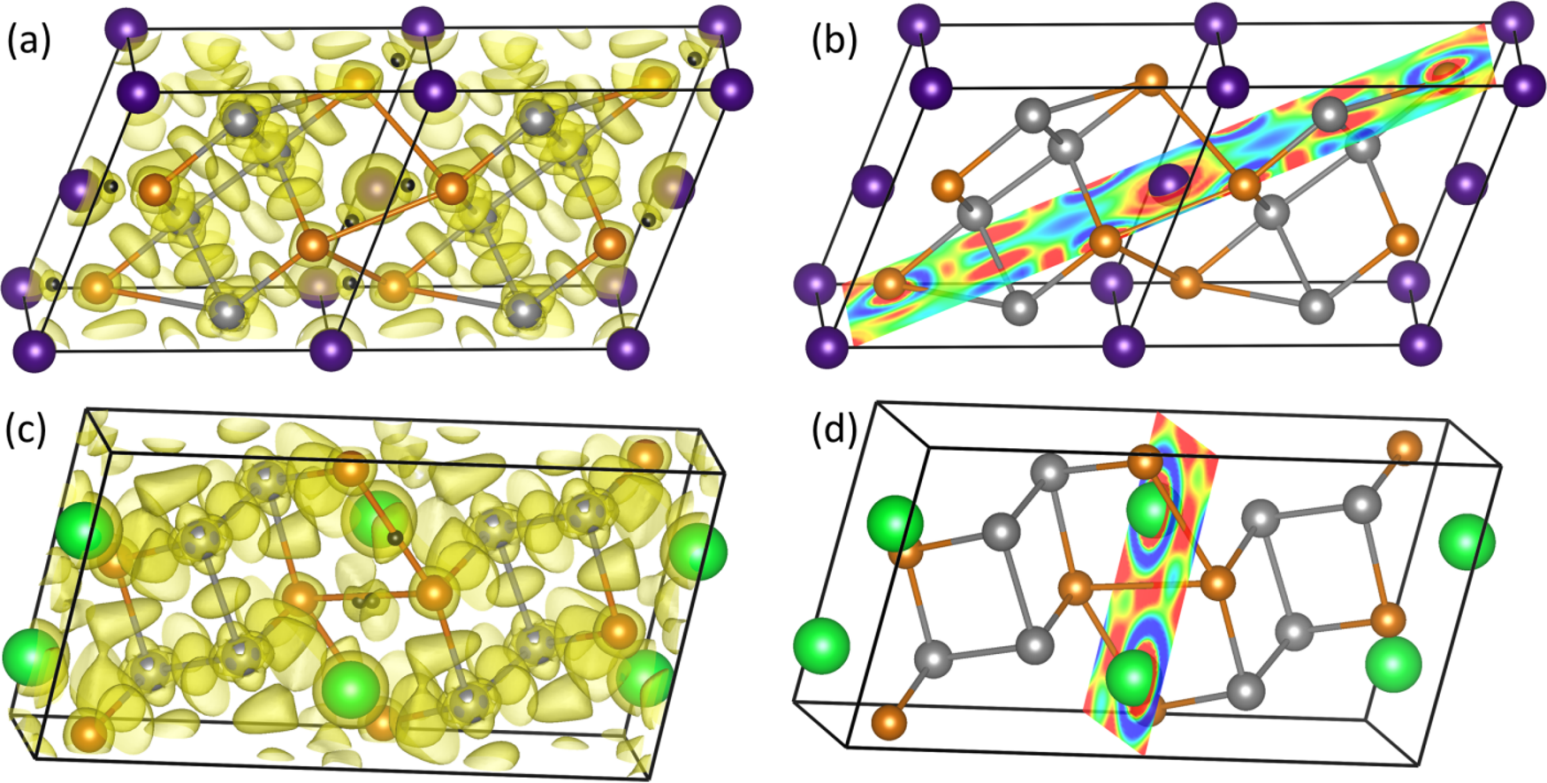
ELF 3D isosurface plot (η = 0.40) in (a) ″PbTaV2″ and (c) ″SrTaV2″ metallic sublattices. ELF attractors are depicted as black spheres. Panels b and d show the 2D-ELF contour plot along the plane containing the attractors and Pb or Sr atoms, respectively. Red regions indicate values of the ELF field higher than 0.40, whereas blue regions correspond to ELF values lower than 0.1. (Color codes are essentially the same as for Figures 2 and 6.)

An illustrative view of the structure of these two compounds relies on the anions in the metallic matrices model (AMM),^41,42^ which explains the final crystalline lattice as a subarray of metal atoms filled by nonmetallic oxygen atoms in positions governed by the electronic structure of this metallic subarray. When the electron density analysis on the metallic ″TaV″ subarrays extracted from the experimental *A*Ta_2_V_2_O_11_ (A = Sr, Pb) *Cc* and *C*2 structures is performed, one interesting result is obtained. At the position where Sr and Pb are located in these compounds, there appear cage points where the ELF and the electron density are minima in both TaV subarrays, thus identifying preferential lattice positions for atoms willing to lose electrons (see Figure S5, electron density). The values of the electron density are as low as ∼1 × 10^–3^. and ∼9 × 10^–4^ a.u. for the *Cc* and *C*2 structures, respectively, which are consistent with the larger size of the *C*2 lattice. If we repeat the ELF analysis on ″*A*Ta_2_V_2_″ subarrays extracted from the experimental *A*Ta_2_V_2_O_11_ (A = Sr, Pb) *Cc* and *C*2 structures, two other interesting results are obtained. In the Sr-based subarray, there are V···V ELF attractors, whereas V···Sr attractors are missing. The other way around is found for the Pb-based subarray. The existence or not of V···V (and A–V) ELF attractors is one signature differentiating these two structures. As we anticipate above, the ELF 3D isosurfaces and 2D counter plots reveal particular distortions of the electron distribution around Pb atoms when compared with the picture of Sr atoms in their respective structures (see Figure 8a–d).

To verify that the differences in the ELF picture between the two compounds are not due to their crystalline structures, we calculate the ELF topology of the ″PbTa2V2″ subarray extracted from a hypothetical PbTa_2_V_2_O_11_*Cc* structure with the same unit cell as the one found in the SrTa_2_V_2_O_11_ (*Cc*) compound. The ELF analysis shows the same distinctive peculiarities as in the canonical PbTa_2_V_2_O_11_*C*2 structure. The distortion of the Pb electron density is a genuine feature that is related to the lone pair activity of this atom and is not induced by the particular *C*2 lattice. Following O’Keeffe and Hyde,^43^ the interactions between metallic atoms lead to low energetic contributions in ionic crystals but are important enough to determine the final stability of the structure. Therefore, we can understand the structural differences between the Pb and Sr structures as due to the existence of residual Pb···V metallic interactions in the PbTVO compound drawn by the anisotropic electron distribution of the Pb lone pair as shown in Figures 2f and 8b,d.

Generally, the vanadium-incorporated SBT demonstrates enhanced dielectric properties^44,45^ reaching the extremum at the Curie temperature. We accessed the dielectric constants at their static limit for an ideal single crystal at *T* = 0 K, and those parameters were practically the same (ε_rSTVO_ = 21 ≈ *ε*_rPTVO_ = 22). We note that the obtained values are lower than those detected for SBT compositions enriched with vanadium content^45^ probed at a low frequency and close to the room temperature limit. Yet, it is known that the peak dielectric constant of SrBi_2_(Ta_2–*x*_V_*x*_)O_9_ ceramics is the higher the state of doping for a wide range of temperatures; therefore, corresponding measurements are worth to consider as a perspective for future studies.

## 4 Conclusions

The subtle structural distortions are intrinsic for *A*Ta_2_V_2_O_11_ (A = Sr or Pb) due to polarized Ta–O and V–O bonds that stabilize the polar lattice of monoclinic symmetry for both compounds, in contrast to previous studies, as revealed by high-resolution X-ray diffraction.^6,11,13^ The chemical type of the counter-cation, Sr or Pb in the present case, has further influence on the overall crystal packing via the local oxygen environment built up around them in a specific fashion. The atomistic view projected on studied compounds helps us to formulate several outcomes: (i) Pb 6s^2^ participates in the construction of the top of valence band zone in contrast to the Sr analogue; (ii) the AMM approach allows one to detect residual V Pb interactions induced by the anisotropic behavior of the lone pair in the interior of Pb metallic centers. Anomalies in Born charges of strontium (lead), tantalum, and structurally related oxygen atoms are similar to some of layered perovskite-type ferroelectrics, while vanadium species are inactive in the formation of the polar structure of STVO and PTVO. Calculated band gaps are found to be consistent with experimental data of ref (11), although these are relatively far from the desirable 1.3–1.6 eV for photovoltaic applications. This implies that careful doping of parental matrices with proper cations is a suitable approach to narrow the band gaps and may pave the way for further studies of bulk photovoltaic effects of tuned material.

## Authors Contributions

A.A.B. conceived and designed the project. I.V.O. synthesized the materials and carried out thermo-analytical measurements. A.A. performed the electron microscopy inspection and analyzed the HRTEM and SAED results. A.A.B. and S.G.-G. analyzed the X-ray diffraction data. A.A.B, A.L., and J.M.R. developed the computational part of the study. All authors discussed the results and contributed to writing and revising the manuscript.

## Supporting Information Available

Crystallographic summary and table of bond lengths: SrTa_2_V_2_O_11_ (CIF)
Crystallographic summary and table of bond lengths: PbTa_2_V_2_O_11_ (CIF)
The traces of (*D*)TG scans and the transcription of thermal effects; the final Rietveld refinement plots; supplementary HRTEM and related SAED images; equilibrium coordinates of DFT-relaxed crystal structures; principal components of Born charge and dielectric tensors; and diagrams of the electron density section of metallic subarrays for STVO and PTVO (PDF)

## Supplementary Material

jp2c00469_si_001.cif

jp2c00469_si_002.cif
