# Supplementary material for: Structural and Electronic Effect Driven Distortions in Visible Light Absorbing Polar Materials ATa2V2O11 (A = Sr, Pb)
Source: J Phys Chem C Nanomater Interfaces. 2022 Apr 28;126(18):8047–55. doi: 10.1021/acs.jpcc.2c00469 (PMC9109282; doi:10.1021/acs.jpcc.2c00469)
Supplement: Supplementary file 3 — jp2c00469_si_003.pdf [file jp2c00469_si_003.pdf]

Structural and Electronic Effect Driven Distortions in Visible Light Absorbing Polar Materials $ATa_2V_2O_{11}$ (A –Sr, Pb)

Artem A. Babaryk,[a*] *Ievgen V. Odynets,*[b†] *Álvaro Lobato,*[c] *Alaa Adawy,*[d] *J. Manuel Recio*[e*] *and Santiago Garcia-Granda*[a]

[a] Department of Physical and Analytical Chemistry, University of Oviedo – CINN (CSIC), 33006 Oviedo, Spain
[b] Taras Shevchenko National University of Kyiv, 64/13 Volodymyrska St., Kyiv 01601, Ukraine
[c] Malta-Consolider Team and Departamento de Química Física, Universidad Complutense de Madrid, 28040 Madrid, Spain
[d] Unit of Electron Microscopy and Nanotechnology, Institute for Scientific and Technological Resources (SCTs), University of Oviedo, 33006 Oviedo, Spain
[e] MALTA-Consolider Team and Departamento de Química Física y Analítica, Universidad de Oviedo, 33006 Oviedo, Spain

*Corresponding author: *Artem A. Babaryk*. E-mail address: babarykartem.uo@uniovi.es
*Corresponding author: *J. Manuel Recio*. E-mail address: jmrecio@uniovi.es

Table of Contents

Sections	Titles	Pages
I	Thermoanalytical examination of stoichiometric reaction blends	S2–S3
II	Results of Rietveld refinements	S4–S6
III	Supplementary HRTEM and SAED images	S6–S8
IV	Results of DFT calculations at periodic boundary conditions	S9–S10
V	Electron density analysis in submetallic arrays	S11
VI	The list of references	S11

[†] Author's present address: ShimUkraine LLC, Kyiv, Ukraine

Section I

Supporting Information. **Thermoanalytical examination of stoichiometric reaction blends**.

Integrally, a synthesis of $SrTa_2V_2O_{11}$ ($PbTa_2V_2O_{11}$) powder *via* solid state route can be expressed by the equations (1-2):

$$SrCO_{3(s)}+Ta_2O_{5(s)}+2NH_4VO_{3(s)} \rightarrow SrTa_2V_2O_{11(s)}+2NH_{3(g)}+CO_{2(g)}+H_2O(s) \quad (1)$$

$$PbO_{(s)}+Ta_2O_{5(s)}+2NH_4VO_{3(s)} \rightarrow PbTa_2V_2O_{11(s)}+2NH_{3(g)}+H_2O(s) \quad (2)$$

However, as this equation is schematic and it does not provide much information about the optimal temperature for samples preparation. Therefore, a thermal analysis of the stoichiometric initial blends was performed to assess synthetic conditions. 17.43 mg (16.5 mg) of fine-ground mixture of $SrCO_3$ (PbO), Ta_2O_5 and NH_4VO_3 (1:1:2 molar ratio) was loaded into cylindrical Pt/Rh-cruicible and heated up from 25 to 1000 °C at a rate of 10 °C min^{-1} in a flow of air (100 ml min^{-1}) using a Shimadzu DTG-60H simultaneous thermogravimetry/differential thermal analyzer. The changes in a heat flow (DTA) and weight loss (TG, DTG) relatively to Al_2O_3 were simultaneously recorded. Summarizing details of this study are briefed in the Tables S1 and S2. The major weight loss takes place in a temperature range 140–600 °C over three sequential stages (Table S1). At the first stage the weight loss (6.82 wt.%) is ascribed to a release of ammonia and water molecules under NH_4VO_3 decomposition [Biedunkiewicz, 2012]. Following weight loss (5.51 wt. %) is observed in a temperature range of 340–645 °C due to the CO_2 extrusion caused by the interaction between thermally activated strontium carbonate and vanadium oxide formed at previous stage. In the temperature range 645–1000 °C (stage III), the negligible weight loss of only 0.83 wt. % is detected corresponding to small endothermic peak appears on DTA curve at 675 °C (Figure S1). Accoding to the reference data [Brown, Jr. 1972], strontium dimetavanadate melts congruently close to 645 °C. Finally, the formation of target product spans over 800°C–950 °C, although this process cannot be followed on the DTA curve, likely, because a reactant mixture contains high-melting and slowly-reacting tantalum (V) oxide. Thus, the reaction dwell is a crucial factor for the prepation of STVO under the strict temperature control. Further heating to 997 °C does not improve the synthetic conditions and results in discomposure of the compound. Therefore, a survey of thermoanalytical data suggests the reaction temperature of 800 °C is enough to prepare target compound using conventional solid-state reaction technique.

While the application of similar conditions for "$PbTa_2V_2O_{11}$" blend anticipatively results in Stage I at lower temperatures, a replacement of $SrCO_3$ for PbO directly tranforms the Stage II. The sharp exo-effect at 491 °C is possible due to incongruent melting of PbV_2O_6 [Viting, 1964]. At elevated temperatures the pronounced peak can be observed centered at 837 °C. Found value is very close to that was found previously by Paidi et al. (ca .815 °C) from differential scanning calorimetry [Paidi, 2017].

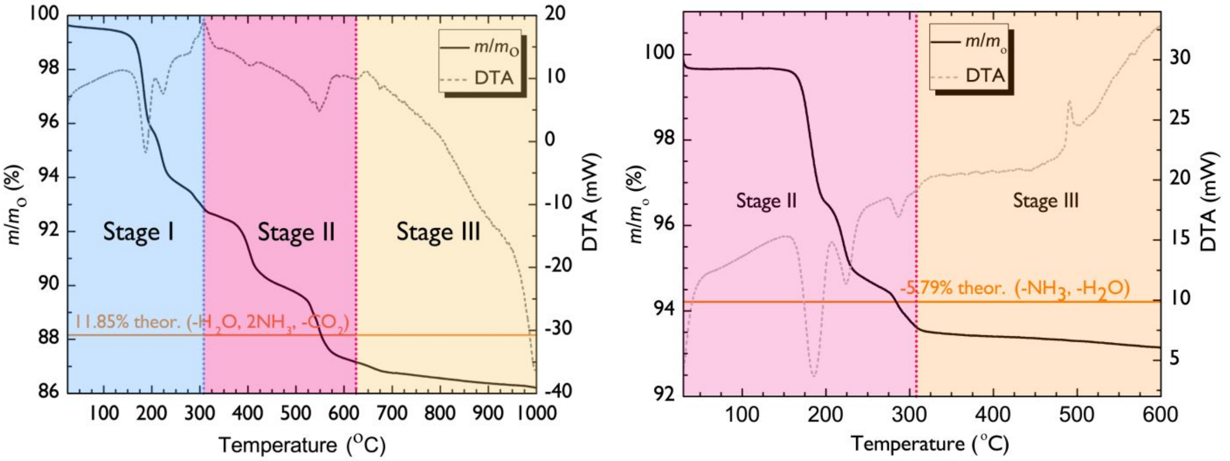

Figure S1. TG and DTA traces of "$SrTa_2V_2O_{11}$" (left) and "$PbTa_2V_2O_{11}$" (right) reacting blend after heating in air flow.

Table S1. Summary of thermoanalytical data for $SrCO_3$(PbO)/NH_4VO_3/Ta_2O_5 (1:2:1) reactive blend.

Stage	TG		DTG	DTA	
	$\Delta(m/m_o)_{exp}$, %	$\Delta(m/m_o)_{theor}$, %	T_{peak}, °C	T_{peak}, °C	Effect type
I	6.82(6.15)[a]	6.51(5.79)	182(180)<br>218(222)<br>285(283)	187(186)<br>223(224)<br>308(287)	*endo*<br>*endo*<br>*exo*(*endo*)
II	5.51	5.34	397<br>523<br>547<br>–	406<br>532<br>548<br>625	*endo*
III	0.83(0.39)	–(–)	–(–)	675(491)<br>997	*endo*(*exo*)

[a]The second figure given parentheses is for $PbTa_2V_2O_{11}$

Table S2. Tentative assignment of dominant processes over scanned temperature region.

Stage	Reaction Equation	Temperature Ranges
I	$2NH_4VO_3 \rightarrow 2NH_3\uparrow + V_2O_5 + H_2O$	140–340 °C
II	$SrCO_3 + V_2O_5 \rightarrow SrV_2O_6 + CO_2\uparrow$<br>($PbO + V_2O_5 \rightarrow PbV_2O_6$)	340–645 °C<br>(340–485 °C)
III	$Sr(VO_3)_2 + Ta_2O_5 \rightarrow SrTaV_2O_{11}$	800–950 °C

Section II

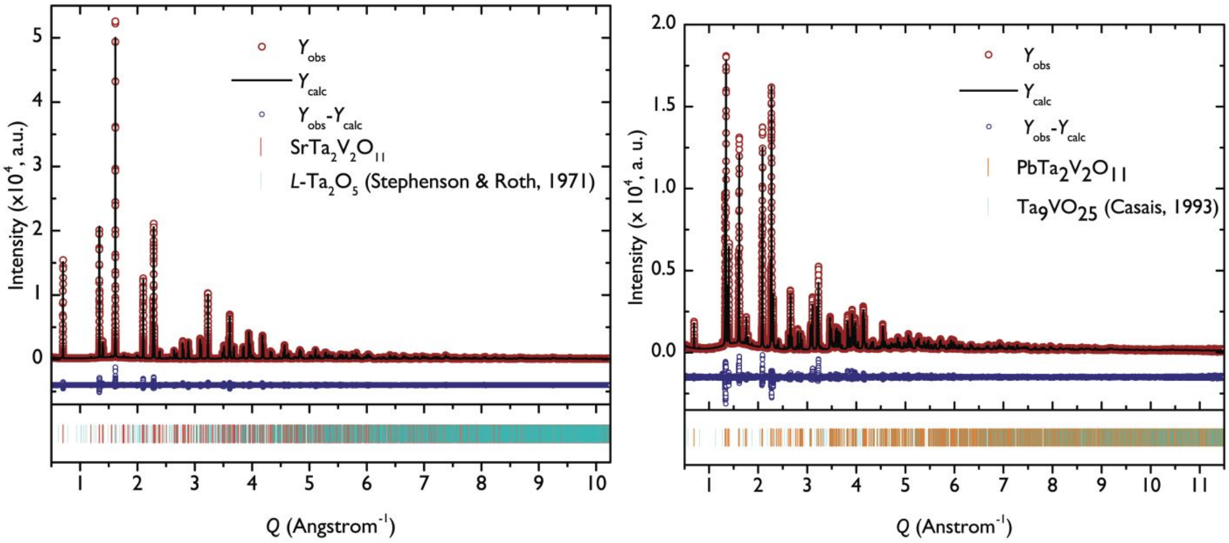

Figure 2. Rietveld-refined SR-PXRD patterns of STVO (left panel) and PTVO (right panel).

Table S3. Selected parameters of the Rietveld refinements of the $SrTa_2V_2O_{11}$ structure from SR-PXRD data (APS 11BM-B, IL, USA).

T (K)	293
Space group	Cc
a, b, c (Å)	18.17028(1), 5.52870(1), 9.52248(1)
β (deg)	99.81446(13)
Z	4
Cell volume (Å^3)	942.6098(23)
Calcd density (g cm^{-3})	5.12562(1)
Radiation wavelength (Å)	0.457904
^aR_p, $^bR_{wp}$, $^c\chi^2$	0.055, 0.070, 1.262

$^aR_p = (\Sigma|Y_{o,m}$-$Y_{c,m}|)\ (\Sigma Y_{o,m})^{-1}$; $^bR_{wp} = \{\Sigma w_m(Y_{o,m}$-$Y_{c,m})^2\ (\Sigma w_m Y_{o,m}{}^2)\}^{1/2}$; $^c\chi^2=\{w_m(Y_{o,m}$-$Y_{c,m})^2\ (M - P)^{-1}\}^{1/2,}$ where where $Y_{o,m}$ and $Y_{c,m}$ are the observed and calculated data respectively at data point m, M the number of data points, P the number of parameters, wm the weighting given to data point m which for counting statistics is given by $w_m=\sigma(Y_{o,m})^{-2}$ where $\sigma(Y_o,m)$ is the error in $Y_{o,m.}$

Table S4. Selected interatomic distances for the $SrTa_2V_2O_{11}$ structure.

Bond	Distance	BVS	Bond	Distance	BVS
		Ta–O bonds			
Ta1–O2	1.870(19)	1.13	Ta2–O7	1.840(21)	1.23
Ta1–O3	1.886(21)	1.08	Ta2–O3	1.874(20)	1.12
Ta1–O7	1.948(18)	0.92	Ta2–O2	1.932(20)	0.96
Ta1–O1	2.017(11)	0.76	Ta2–O10	1.974(19)	0.86
Ta1–O9	2.119(19)	0.58	Ta2–O6	2.028(16)	0.74
Ta1–O5	2.210(20)	0.45	Ta2–O8	2.090(19)	0.63
		V–O bonds			
V1–O5	1.588(20)	1.77	V2–O11	1.517(22)	2.14
V1–O4	1.640(21)	1.54	V2–O8	1.686(20)	1.36
V1–O1	1.7167(87)	1.25	V2–O6	1.748(13)	1.15
V1–O9	1.719(21)	1.24	V2–O10	1.802(20)	1.00
		Sr–O bonds			
Sr1–O9	2.541(22)	0.21	Sr1–O8	2.733(21)	0.12
Sr1–O10	2.604(20)	0.18	Sr1–O4	2.759(13)	0.12
Sr1–O6	2.630(14)	0.16	Sr1–O11	2.760(22)	0.12
Sr1–O1	2.721(11)	0.13	Sr1–O5	2.774(21)	0.11
Sr1–O11	3.087(16)	0.05			

Table S5. Selected parameters of the Rietveld refinements of the $PbTa_2V_2O_{11}$ structure from SR-PXRD data (APS 11BM-B, IL, USA).

T (K)	293
Space group	*C*2
a, *b*, *c* (Å)	9.63822(3), 5.44711(2), 9.61842(3)
β (deg)	109.35836(22)
Z	2
Cell volume (Å^3)	476.4221(25)
Calcd density (g cm^{-3})	5.90415(3)
Radiation wavelength (Å)	0.457904
^aR_p, $^bR_{wp}$, $^c\chi^2$	0.063, 0.079, 1.574

$^aR_p = (\Sigma|Y_{o,m}-Y_{c,m}|)\ (\Sigma Y_{o,m})^{-1}$; $^bR_{wp} = \{\Sigma w_m(Y_{o,m}-Y_{c,m})^2\ (\Sigma w_m Y_{o,m}{}^2)\}^{1/2}$; $^c\chi^2=\{w_m(Y_{o,m}-Y_{c,m})^2\ (M - P)^{-1}\}^{1/2,}$ where where $Y_{o,m}$ and $Y_{c,m}$ are the observed and calculated data respectively at data point *m*, *M* the number of data points, *P* the number of parameters, *wm* the weighting given to data point *m* which for counting statistics is given by $w_m=\sigma(Y_{o,m})^{-2}$ where $\sigma(Y_o,m)$ is the error in $Y_{o,m.}$

Table S6. Selected interatomic distances for the $PbTa_2V_2O_{11}$ structure

Bond	Distance	BVS	Bond	Distance	BVS
		Pb–O bonds			
Ta1–O3	1.8175(90)	1.31	Ta1–O6	1.9871(74)	0.83
Ta1–O5	1.9048(19)	1.03	Ta1–O1	2.0840(70)	0.64
Ta1–O3	1.9613(91)	0.89	Ta1–O4	2.2000(75)	0.46
		V–O bons			
V1–O4	1.5652(77)	1.88	V1–O1	1.6845(71)	1.36
V1–O2	1.5802(69)	1.81	V1–O6	1.7486(79)	1.15
		Pb–O bonds			
Pb1–O2	2×2.6269(78)	0.20	Pb1–O1	2×2.6762(51)	0.18
Pb1–O6	2×2.6757(88)	0.18	Pb1–O4	2×2.9555(89)	0.08

Section III

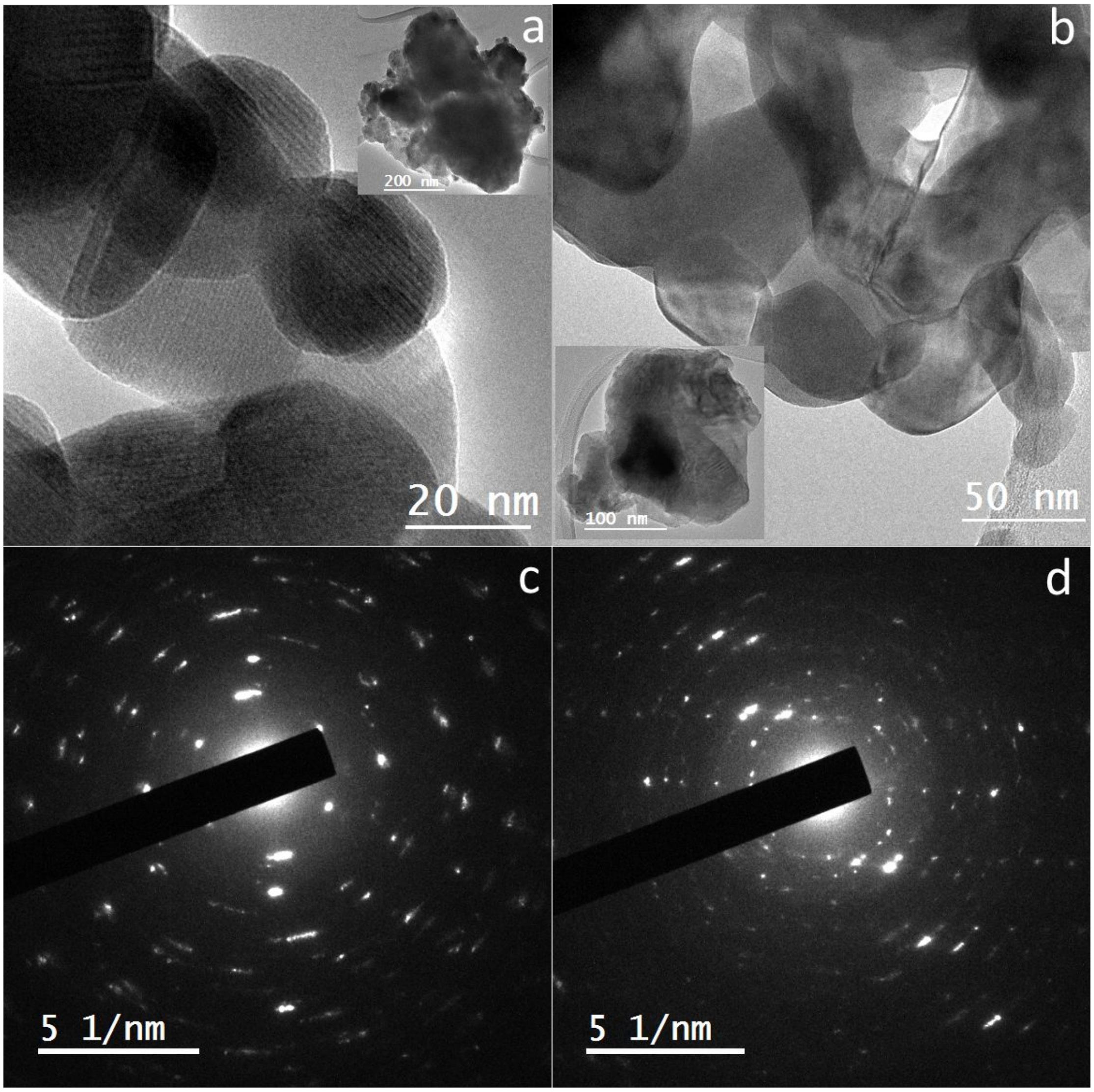

Fig S3. TEM images of $SrTa_2V_2O_{11}$ (a) and $PbTa_2V_2O_{11}$ (b) and the corresponding SAED ring patterns of $SrTa_2V_2O_{11}$ (c) and $PbTa_2V_2O_{11}$ (d)

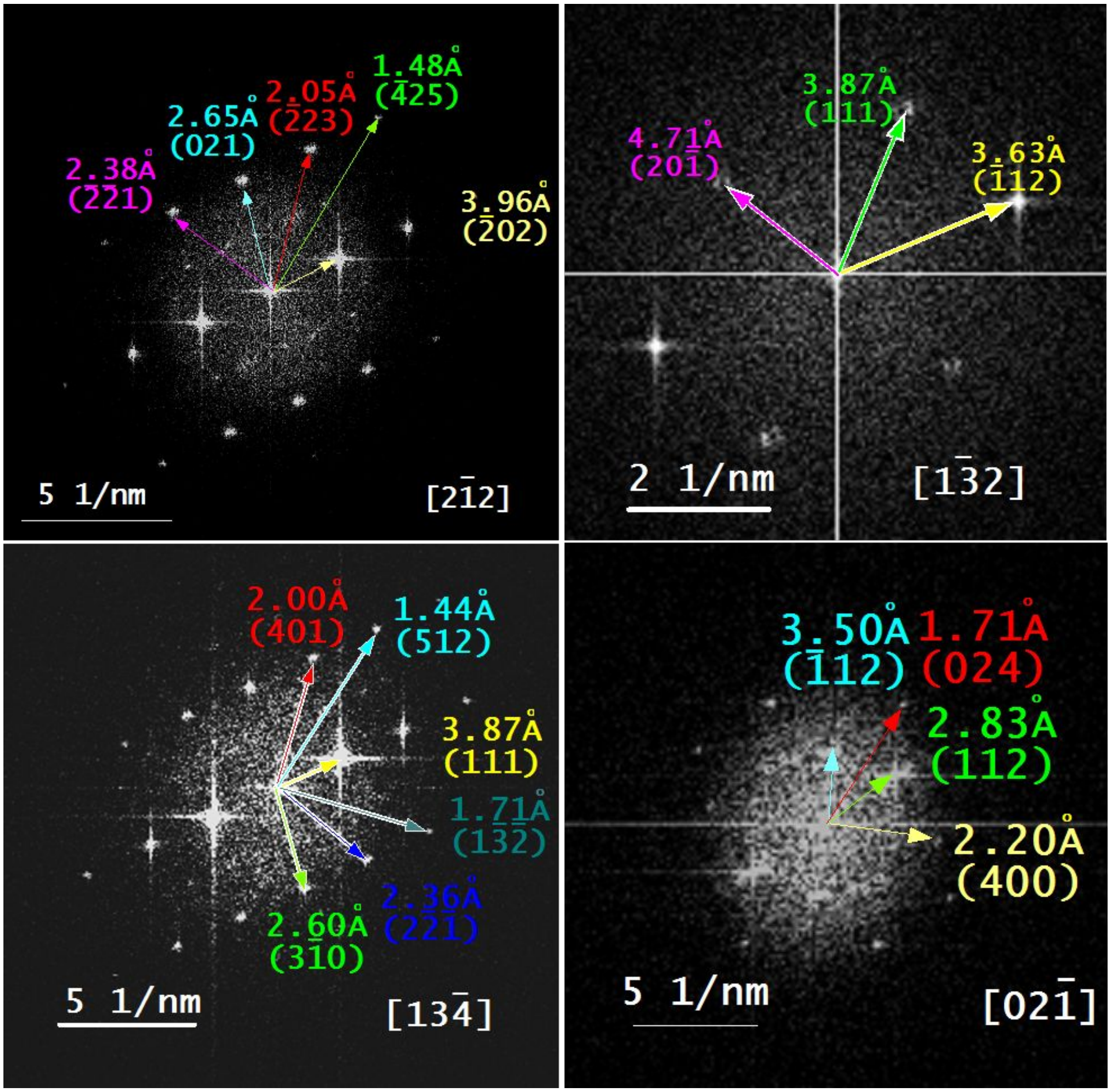

Fig S4. Indexed fast Fourier transforms for $PbTa_2V_2O_{11}$ at different zone axes

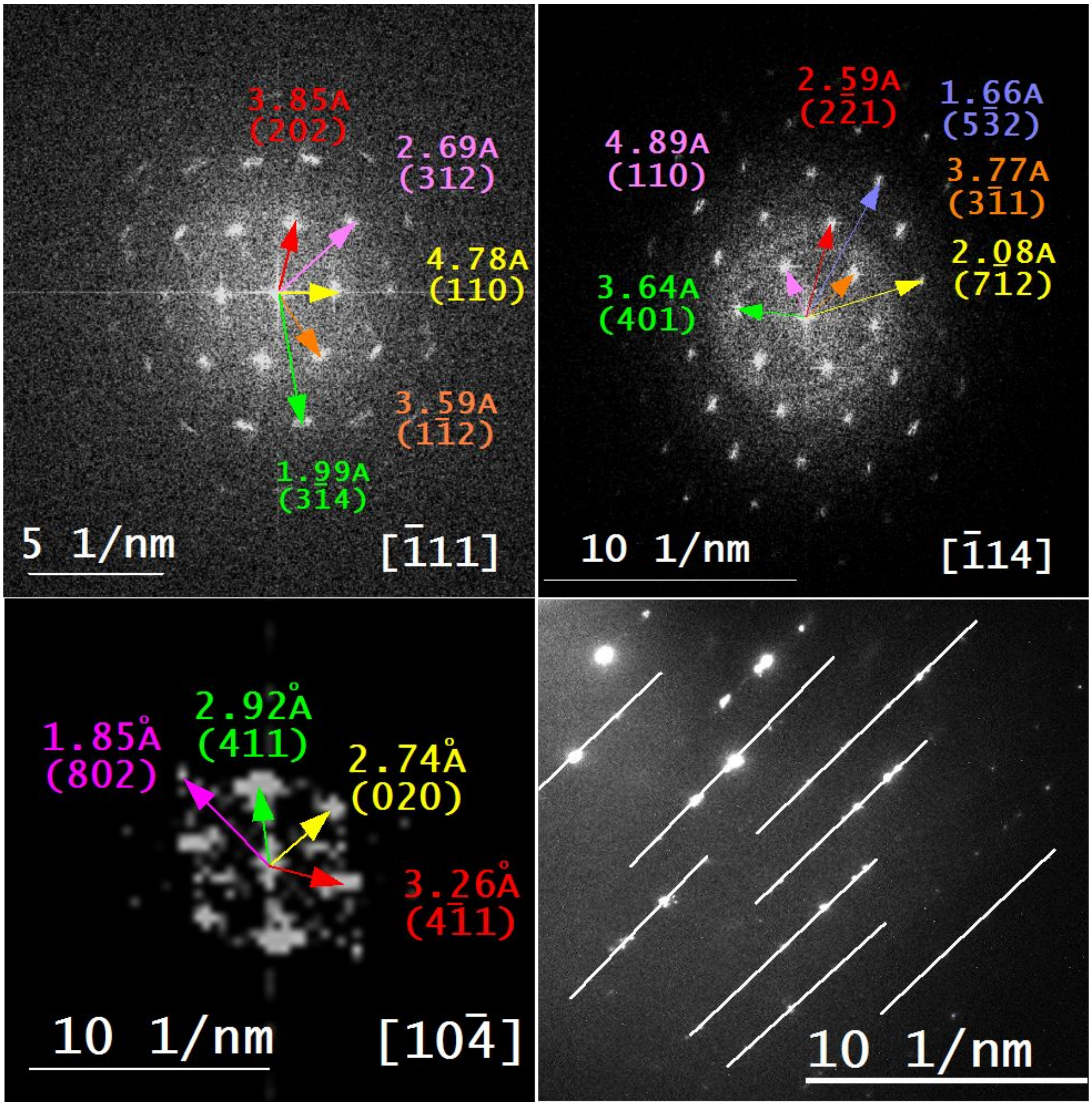

Fig. **S5**. Indexed fast Fourier transforms for $SrTa_2V_2O_{11}$ at different zone axes and a SAED pattern in the zone axis [100] showing the short-range directional ordering of the plane (001) as further demonstrated in Fig. 5C.

Section IV

Table S7. The unit cell parameters and fractional atomic coordinates of $SrTa_2V_2O_{11}$ after structure relaxation.

***Space group – Cc, a* = 18.15761 Å, *b* = 5.53915 Å, *c* = 9.52015 Å, *β* = 99.7554°, *V* = 943.670 Å³**				
Atom	***x*/*a***	***y*/*b***	***z*/*c***	***Hirshfeld Charge***
Ta1	0.806170	0.251430	0.192100	3.05
Ta2	0.693760	0.248450	0.820900	3.04
V1	0.407760	0.236860	0.048080	2.06
V2	0.092130	0.262880	0.940970	2.06
Sr1	0.500170	0.302750	0.716190	1.05
O1	0.380490	0.040010	0.607850	−1.04
O2	0.736960	0.254110	0.017250	−1.17
O3	0.749450	0.029440	0.282860	−1.19
O4	0.497670	0.264550	0.107980	−0.75
O5	0.866850	0.020970	0.620220	−1.01
O6	0.605780	0.038930	0.859770	−1.06
O7	0.763360	0.471260	0.765200	−1.18
O8	0.633000	0.242840	0.618430	−1.01
O9	0.392920	0.252720	0.861760	−1.06
O10	0.121140	0.019330	0.848100	−1.05
O11	0.002420	0.225890	0.942360	−0.75

Table S8. The unit cell parameters and fractional atomic coordinates of $PbTa_2V_2O_{11}$ after structure relaxation.

***Space group – C2, a* = 9.64860 Å, *b* = 5.38030 Å, *c* = 9.68220 Å, *β* = 109.0960°, *V* = 474.967 Å³**				
Atom	***x*/*a***	***y*/*b***	***z*/*c***	***Hirshfeld Charge***
Ta1	0.128200	0.087780	0.391910	3.06
V1	0.391860	0.058960	0.196320	2.08
O1	0.233250	0.019720	0.243140	1.15
O2	0.344280	0.148960	0.022970	−0.91
O3	0.249340	0.369870	0.464620	−1.06
O4	0.501840	0.290280	0.294760	−1.18
O5	0.009550	0.281180	0.783960	−1.19
O6	0.000000	0.162680	0.500000	−1.19
Pb1	0.000000	0.000000	0.000000	1.88

Table S9. The unit cell parameters and fractional atomic coordinates of STVO supergroup structure after structure relaxation.

***Space group – C2/c, a* = 18.13610 Å, *b* = 5.51300 Å, *c* = 9.59310 Å, *β* = 99.6610°, *V* = 945.557 Å³**			
Atom	***x*/*a***	***y*/*b***	***z*/*c***
Ta1	0.693390	0.246410	0.814030
V1	0.908880	0.266920	0.553540
Sr1	0.500000	0.331570	0.750000
O1	0.608820	0.050150	0.877490
O2	0.759100	0.459960	0.737980
O3	0.618040	0.248140	0.629230
O4	0.870260	0.032590	0.637670
O5	0.999400	0.217670	0.587980

O6	0.750000	0.250000	0.000000

Table S10. The unit cell parameters and fractional atomic coordinates of PTVO supergroup structure after structure relaxation.

***Space group – R-3m, a = b* = 5.56240 Å, *c* = 26.9290 Å, *V* = 721.565 Å³**			
Atom	***x*/*a***	***y*/*b***	***z*/*c***
Ta1	0.000000	0.000000	0.128760
V1	0.000000	0.000000	0.392570
Pb1	0.000000	0.000000	0.000000
O1	0.505950	0.494050	0.254610
O2	0.000000	0.000000	0.331780
O3	0.500000	0.000000	0.500000

Table S11. Diagonal elements of the calculated Born effective charge tensor and its average ($Z^*_{avr} = \{Z^*_{xx} + Z^*_{yy} + Z^*_{zz}/3\}$) for the STVO. The nominal charge of each atom (Z_{nom}) is reported for comparison.

Atom	Z^*_{xx}	Z^*_{yy}	Z^*_{zz}	Z^*_{avg}	Z_{nom}
Ta1	7.25	8.88	8.89	8.34	5
Ta2	7.28	8.84	8.87	8.33	5
V1	3.59	4.59	4.52	4.23	5
V2	3.61	4.45	4.67	4.24	5
Sr1	2.89	2.55	2.60	2.68	2
O1	−3.29	−2.99	−1.60	−2.62	−2
O2	−1.61	−1.27	−6.40	−3.09	−2
O3	−1.55	−5.76	−1.98	−3.10	−2
O4	−2.22	−1.28	−1.01	−1.50	−2
O5	−1.33	−4.75	−1.59	−2.56	−2
O6	−1.57	−4.55	−1.71	−2.61	−2
O7	−1.59	−5.71	−1.93	−3.08	−2
O8	−1.30	−0.96	−5.40	−2.55	−2
O9	−1.57	−1.05	−5.20	−2.61	−2
O10	−3.34	−2.80	−1.74	−2.63	−2
O11	−2.44	−1.09	−0.97	−1.50	−2

$\varepsilon_{xx} = 7.98$, $\varepsilon_{yy} = 26.31$, $\varepsilon_{zz} = 27.77$

Table S12. Diagonal elements of the calculated Born effective charge tensor and its average ($Z^*_{avr} = \{Z^*_{xx} + Z^*_{yy} + Z^*_{zz}/3\}$) for the PTVO. The nominal charge of each atom (Z_{nom}) is reported for comparison.

Atom	Z^*_{xx}	Z^*_{yy}	Z^*_{zz}	Z^*_{avg}	Z_{nom}
Ta1	8.66	8.96	7.62	8.41	5
V1	4.77	4.63	3.43	4.28	5
Pb1	3.50	3.17	3.70	3.46	2
O1	−0.89	−5.26	−2.27	−2.81	−2
O2	−1.45	−0.99	−2.64	−1.69	−2
O3	−4.42	−2.37	−2.54	−3.11	−2
O4	−3.70	−1.86	−1.94	−2.50	−2
O5	−4.06	−1.95	−2.30	−2.77	−2
O6	−1.30	−5.70	−2.53	−3.18	−2

$\varepsilon_{xx} = 18.01$, $\varepsilon_{yy} = 24.12$, $\varepsilon_{zz} = 24.69$

Section V

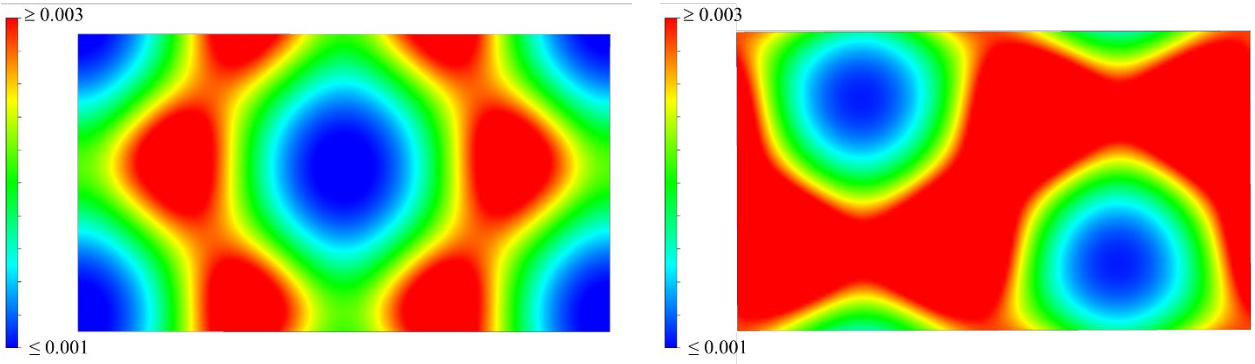

Figure S5. Electron density 2D heatmap for the PTVO-TaV submetallic array along the (001) plane (left) and STVO-TaV submetallic array along the (100) plane (right). Dark blue regions correspond to electron density cage points.

Section VI

References

S1 Biedunkiewicz, A.; Gabriel, U.; Figiel, P.; Sabara, M. Investigations on NH_4VO_3 Thermal Decomposition in Dry Air. *J. Therm. Anal. Calorim.* **2012,** *108*, 965–970.

S2 Brown Jr., J.J. Phase Equilibria in the System SrO–CdO–V_2O_5. *J. Am. Ceram. Soc.* **1972**, *55*, 500–503.

S3 Viting, L.M.; Golubkova, G.P. *Vestn. Mosk. Univ., Ser. 2: Khim.* **1964**, *19*, 88–89.

S4 Paidi, A. K.; Jaschin, P. W.; Varma, K. B. R.; Vidyasagar, K. Syntheses and Characterization of $AM_2V_2O_{11}$ (A = Ba, Sr, Pb; M = Nb, Ta) Vanadates with Centrosymmetric and Noncentrosymmetric Structures. *Inorg. Chem.* 2017, *56*, 12631–12640.
